# Dual-cycle dielectrophoretic collection rates for probing the dielectric properties of nanoparticles

**DOI:** 10.1002/elps.201200422

**Published:** 2013-03-06

**Authors:** David J Bakewell, David Holmes

**Affiliations:** 1 Department of Electrical Engineering and Electronics, University of LiverpoolLiverpool, UK; 2 London Centre for Nanotechnology, University College LondonLondon, UK

**Keywords:** AC electrokinetics, Amplitude modulation, Dielectrophoretic spectroscopy, Nanoparticle characterization, Pulsed dielectrophoresis

## Abstract

A new DEP spectroscopy method and supporting theoretical model is developed to systematically quantify the dielectric properties of nanoparticles using continuously pulsed DEP collection rates. Initial DEP collection rates, that are dependent on the nanoparticle dielectric properties, are an attractive alternative to the crossover frequency method for determining dielectric properties. The new method introduces dual-cycle amplitude modulated and frequency-switched DEP (dual-cycle DEP) where the first collection rate with a fixed frequency acts as a control, and the second collection rate frequency is switched to a chosen value, such that, it can effectively probe the dielectric properties of the nanoparticles. The application of the control means that measurement variation between DEP collection experiments is reduced so that the frequency-switched probe collection is more effective. A mathematical model of the dual-cycle method is developed that simulates the temporal dynamics of the dual-cycle DEP nanoparticle collection system. A new statistical method is also developed that enables systematic bivariate fitting of the multifrequency DEP collection rates to the Clausius–Mossotti function, and is instrumental for determining dielectric properties. A Monte-Carlo simulation validates that collection rates improve estimation of the dielectric properties, compared with the crossover method, by exploiting a larger number of independent samples. Experiments using 200 nm diameter latex nanospheres suspended in 0.2 mS/m KCl buffer yield a nanoparticle conductivity of 26 mS/m that lies within 8% of the expected value. The results show that the dual-frequency method has considerable promise particularly for automated DEP investigations and associated technologies.

## 1 Introduction

DEP is an important electrokinetic technique for micromanipulating and transporting micro- and nanoscale biological particles suspended in aqueous media 1–3. DEP is the translational movement of an electrically polarizable body by the action of a nonuniform electric field. It is often implemented by applying radio frequency electrical potentials to microfabricated electrodes immersed in liquid (typically of low conductivity). Biological particles amenable to DEP manipulation include: cells, viruses, nucleic acids (DNA and RNA), proteins, etc.

One of the important applications of DEP is determining dielectric properties of small volume samples. Crossover measurements have been a standard method for characterizing cells, viruses and colloidal bioparticles 4–8. This method often involves preparation of suspension media for a range of controlled conductivities in order to infer a dielectric property, e.g. surface conductivity. Typically, after the sample has been suspended in a suitable DEP chamber, the applied signal is varied until the samples shows a transition from positive to negative DEP (pDEP to nDEP). The signal frequency at transition (i.e. the zero force point, where the polarizability of the particle equals that of the suspending medium) is the recorded crossover frequency for that particular medium conductivity. Unfortunately, the DEP crossover technique requires (i) the existence of both pDEP and nDEP, (ii) substantial time to prepare a range of medium conductivities and perform experiments, (iii) substantial number of specimen samples needed for each crossover experiment, and (iv) the need for considerable operator skill and avoidance of error.

An alternative method for determining the dielectric properties of particles is the initial collection rate. The rate of accumulation of samples into specified collection volume is approximately proportional to the DEP force. The rate, therefore, enables the dielectric properties of the particle to be inferred, e.g. by fitting the rates to the frequency-dependent polarizability, which can be modeled by the real part of the Clausius–Mossotti (CM) function. The collection rate technique, coupled with the need to circumvent problems associated with crossover measurements and the popular use of programmable automated switching software, e.g. LabVIEW™, motivates using collection rates as a means of determining dielectric properties of samples.

**Figure 1:**
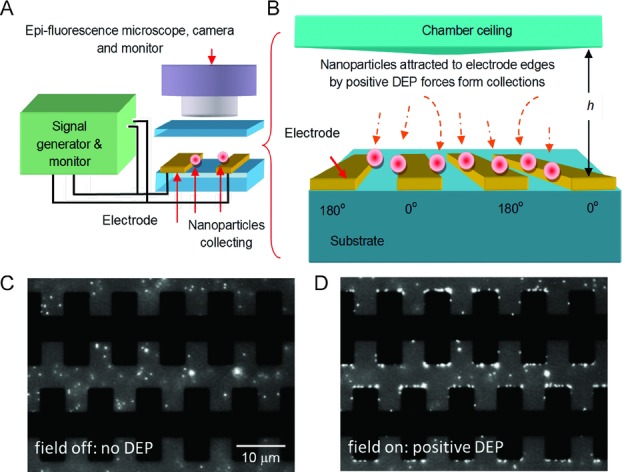
Scheme of the DEP experiment (A) microelectrode array, signal source (including amplifier and monitoring oscilloscope), epifluorescence microscope and camera. (B) DEP collection experiment using interdigitated planar microelectrodes. (C) Fluorescent image showing castellated microelectrodes and 200 nm fluorescent nanospheres without the field applied and (D) with electric field applied showing particles collecting at the electrode edges (i.e. high-field gradient regions) under the influence of pDEP.

A useful method for measuring particle collection rates and inferring dielectric properties is pulsed DEP; measurements have been reported for cells and their constituents, e.g. DNA, RNA, viruses 4,9–17. Figure 1 shows the typical experimental setup for collection rate measurements using planar microelectrodes. Pulsed DEP is the application of DEP for short time durations, typically varying from milliseconds to seconds and the pulse shapes vary from rectangular, ramped, triangular, and so forth. Typically, under the influence of pDEP nanoparticles collect at the edges of the microelectrodes when the signal is switched “on,” and are subsequently released during the “off” phase. Continuously pulsed DEP is also called amplitude modulated (AM) DEP because the pulse physically consists of a sinusoidal waveform with period much less than the pulse duration, and with an amplitude that depends on the shape of the pulse 18.

One of the problems facing the determination of collection rates is that the number of nanoparticles accumulating in the capture region is dependent on the localized concentration at, or slightly above, the planar electrode array—practically this is seen to vary even if the bulk concentration is nominally the same between samples. In previous work using fluorescently labeled particles 12–14, this problem was addressed by normalizing (i.e. taking the ratio) of fluorescence with respect to the initial fluorescence. However, ratio normalization itself can be problematic, particularly when the value of the fluorescence is low. In the regime of low-level fluorescence, it is preferable to adopt alternative methods.

In this paper, we present a novel approach for measuring collection rates and determining the dielectric properties of fluorescent nanoparticles. Our recent research investigating the properties of AM DEP 19 is significantly advanced to allow changing of the carrier frequencies so that between the DEP being switched on, the frequency is switched, or “hops” to a selected frequency suitable for estimating the dielectric properties. The system process is thus titled, dual-cycle DEP. The issue arising from variable nanoparticle concentration localized near the planar array is addressed by using dual frequencies, one as a “control” and the other as a “probe.” The ratio of the “probe” and “control” collection rates is used to infer the dielectric parameters. In addition, frequency-dependent collection rates are often semiquantitatively compared to the real part of the CM function 4,8, fitted by ad hoc multivariate methods 17 and crossover frequencies 16. For the first time, we derive first-principle bivariate statistical algorithms for systematic fitting the real part of the CM using the method of least squares that minimizes residual error.

Clearly, there is considerable motivation for using DEP collection rates for determining dielectric properties of nanoparticles using dual-cycle DEP, yet there is no systematic, quantitative analysis of dual-cycle DEP, or indeed a theoretical framework to utilize this electrokinetic process. This work attempts to remedy this deficiency in the literature by developing mathematical and statistical models for dual-cycle DEP. These models are used to infer the dielectric properties of polymer nanospheres from experimental data.

## 2 Materials and methods

### 2.1 Cyclic DEP nanoparticle transport model

This section introduces and develops a model of a dual-cycle DEP system. The following sections describe the electrokinetic model for nanoparticle collection and release, with new concepts introduced and associated measurement parameters.

#### 2.1.1 Introduction: Single-cycle DEP collection and release

**Figure 2:**
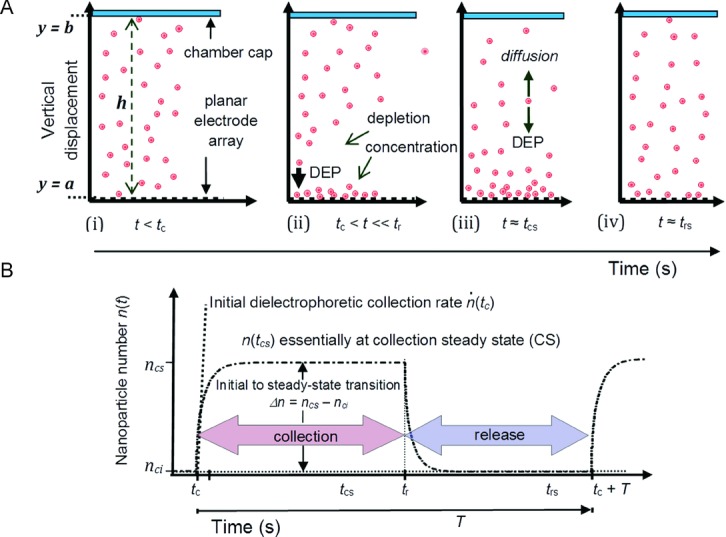
Nanoparticle collection under the action of pDEP force and release after the pDEP force is switched off. (A) Cartoon showing nanoparticle distribution—side view (i) initially uniform (ii) soon after force is switched on (*t* = *t*_c_) pDEP force attracts nanoparticles downwards causing a depletion layer above (iii) depletion layer widens and system reaches collection steady state (*t* ≍ *t*_cs_) where nanoparticle fluxes balance (iv) after the pDEP force is switched off (*t* = *t*_r_) nanoparticles diffuse away from array eventually returning to release SS (*t* ≍ *t*_rs_). (B) Nanoparticle number collection and release within a volume near the array as a function of time showing the initial collection rate and initial to CS transition (further details in text).

A cartoon showing the cyclic movement of nanoparticles attracted by pulsed, or AM, DEP toward and away from a planar electrode array is shown in Fig. 2A. Each cycle with period, *T*, entails a nanoparticle collection phase when DEP is switched “on,” followed by a DEP switched “off” release phase. The distribution of nanoparticles over space and time is described by the nanoparticle concentration, *c*(*y*, *t*); the total number of nanoparticles within the system remains constant throughout the experiment. The corresponding time-dependent nanoparticle number close to the planar electrode array at 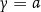 is shown in Fig. 2B. The size of the nanoparticles is on the nanoscale but they can be much larger; the key feature being that the stochastic effect of Brownian thermal motion is significant. The cap is located at height, 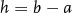 above the array, such that *h* is much greater than the array features.

Before switching on the DEP force at the start of the collection phase of the cycle *t* < *t*_c_, Fig. 2A(i), the nanoparticles are uniformly distributed with the initial number of nanoparticles close to the array, *n*_ci_ (the subscripts “c” and “i” denote “collection” phase of cycle and “initial” time-point). Applying an alternating current potential to the electrodes, the action of the pDEP force causes downward nanoparticle movement, particularly near the electrode array where the DEP force is strong, Fig. 2A(ii). The initial rate of collection, with respect to time, is the nanoparticle collection rate, indicated in Fig. 2B as 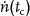. As the concentration further increases near the array, DEP accumulation near the lower boundary results in a depletion layer that steadily rises toward the cap at 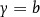. Eventually, the DEP nanoparticle flux becomes balanced by thermally driven diffusion, Fig. 2A(iii), and approaches steady state (SS). Since the SS is for the collection phase, it is denoted “CS” and occurs at time, 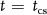, with nanoparticle number, *n*_cs_. Switching off the alternating current potential at 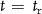 initiates the release phase since there is no longer any pDEP force to trap the nanoparticles, and they diffuse into the bulk medium, Fig. 2A(iv), eventually reaching release SS (release (phase) steady state (RS)) at 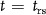. On-off switching can be repeated, as reported for pDEP of DNA and nanospheres 14,19. In the scheme where on-off switch period times are sufficiently long for the system to reach SS in each of the phases, the difference between the collection SS and the initial nanoparticle number close to the array, is the initial to CS transition, 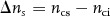. The alternative case when the on-off switch period times are much shorter than the time to SS is considered in the following sections. An important parameter describing the proportion of the time DEP is switched “on” compared to the period, *T*, (or sum of the “on” and “off” durations) is the duty-cycle ratio:


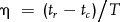
(1)

In Fig. 2B, for example, the duty cycle ratio, η ∼ 0.53.

#### 2.1.2 Dual-cycle system model

**Figure 3:**
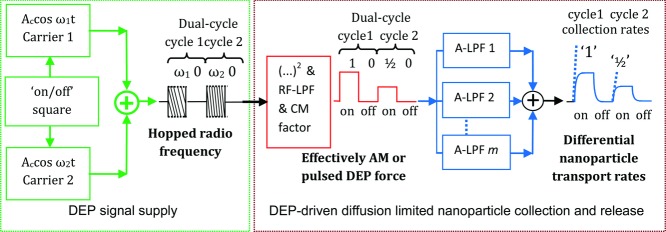
Schematic of the model. The left box shows a switched “on-off” amplitude modulated (AM) generator that frequency hops between two frequencies and produces signal with a rectangular-wave envelope. The signal is fed to the DEP planar array, illustrated previously in Fig. 2(A). The action of DEP process (right box) involves squaring the electric field, effectively removing the radio frequency (RF) carrier by low pass filter (LPF), and the frequency dependence of the real part of the CM factor modulates the pulse amplitude, thus, filtering the signal to a baseband square-wave. The second pulse with amplitude one-half of the first pulse is for illustrative purposes and arises from the CM factor responding less with ω_2_ compared with ω_1_. The output shows nanoparticle transport represented as a series of active ultra-low pass filters (A-LPFs) quantified by the collection rates.

Single cycle DEP collection followed by release has been reported recently 19, here we extend the framework to a dual-cycle entailing two separate collection and release cycles with periods *T*_1_ and *T*_2_ for the first and second cycles, respectively, and total period, 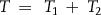. A schematic of the dual-cycle DEP system is shown in Fig. 3. A signal generator, shown in the left box, supplies voltages to the microelectrodes and can switch both signal amplitude and applied frequency. The electrical potential at the output of the *j*th dual-cycle is given by:


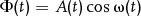
(2)

where 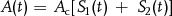 and 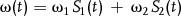. The switch functions *S*_1_ and *S*_2_, applicable for the first and second cycles, are defined as unity (on) for *t* within the specified interval and zero (off) elsewhere:


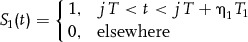
(3)


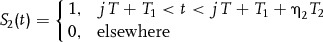
(4)

and they can be easily constructed from Heaviside unit-step functions 20. *A*_c_ is the ground-to-peak cosine amplitude, where the subscript “c” denotes carrier. The two angular frequencies for the first and second cycles are ω_1_ and ω_2_ that the signal generator is programmed to switch or “hop” between, and η_1_ and η_2_ are the respective duty-cycle ratios. For convenience, the dual-cycle period *T* is said to have a modulation frequency 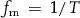 with subscript “m” denoting modulation. The output sinusoidal signal of the generator is shown in the left box of Fig. 3 with a rectangular-wave envelope of the dual cycle; the first cycle with frequency ω_1_ and the second with frequency ω_2_ and the same amplitude as the first.

The DEP force on a nanoparticle located at position 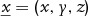 is proportional to the small-time averaged gradient of the square of the electric field magnitude. The spatial variation of the electric field (peak value) can be evaluated from Laplace's equation and written as follows:


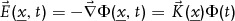
(5)

where the normalized electric field, 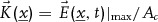, assumes a unit ground-to-peak voltage. The DEP process is represented in the right box of Fig. 3. Using Eqs. (2)–(5), the small-time averaged DEP force on a nanosphere with radius, *r*, immersed in a medium with dielectric constant, ε_m_, is given by:


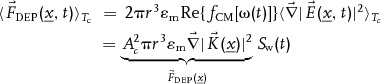
(6)

where the switch function is weighted by the real part of the CM factor, 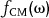, associated with each of the two cycles:


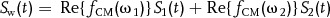
(7)

In Eqs. (6) and (7) 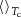 denotes the small-time average over the relevant carrier frequency period, *T*_c_, 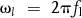 with l being the cycle index, 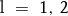, and “Re” denotes real part of the CM function that is bounded, 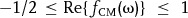. It is evaluated according to each of the carrier frequencies since both 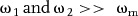 for the main harmonics of the square-wave modulation.

The effect of the real part of the CM function is shown in the right box of Fig. 3 and is included with the square-law and radio frequency carrier low pass filtering effect that represents the physical processes in which the DEP force arises. The CM is frequency dependent because it is a function of the dielectric properties of the nanoparticles suspended in aqueous medium. Hence, the second cycle (of switched frequency) yields a different amplitude, in this example it is half that of the first pulse. The mass of nanoparticles is small and it can be assumed that they reach terminal velocity instantaneously. In order to characterize their transport under the action of continuous on-off pulsed, or AM DEP, the effects of thermally driven Brownian motion are included. The spacetime evolution of concentration, 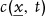, can be written in differential form as the modified diffusion equation (MDE), or Fokker–Planck equation:


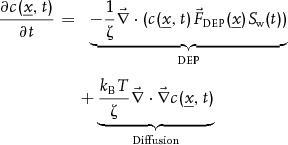
(8)

where *k*_B_ *T* is the Boltzmann temperature and *ζ* is the nanoparticle dynamic drag coefficient. The nanoparticle collection “on” and release “off” processes can be considered separately using the switch functions *S*_1_ and *S*_2_ that rapidly change over the order of nanoseconds. The duration of switching occurs with a timescale that is much smaller than the timescales of interest for the collection and release times. The general solution of a linear MDE, for each of the two phases can be determined using the separation of variables method and yields a Fourier series with exponential time-decay terms 20–22 typical of a diffusion limited process. The linear MDE (Eq. (8)) is applicable for very low to moderate concentrations where the nanoparticles do not interact and can be derived either by stochastic integration of a single particle Langevin equation 23 or by using standard mass continuity methods. In the frequency domain, the series can be represented as a series of active ultralow pass filters as shown in Fig. 3, right box. This means that the rectangular DEP force pulses become rounded with collection rates being approximately proportional to the two cyclic DEP forces, as shown, and their comparison is a differential collection rate.

#### 2.1.3 Dual-cycle time profiles

The initial collection rate can be found by assuming the initial concentration is uniform in Eq. (8) so that 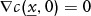 and that the capture volumes are sufficiently small. The rate of capture, for sufficiently dilute, initially uniform concentrations where the nanoparticles do not interact, is approximately proportional to the DEP force, 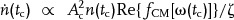 9–14,16. Given that the nanoparticle parameters in Eq. (6) are equal in each of the two cycles except the CM factor, then it follows that the differential rate is the ratio of the second rate divided by the first:


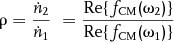
(9)

Important as they are, the collection rates alone cannot characterize the entire time profile for the nanoparticle number that describes the system dynamics. The solution for the nanoparticle number is found by integrating Eq. (8) in space and time and including the switch functions. The expression for the *j*th dual-cycle nanoparticle number is:


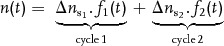
(10)

where 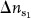 and 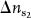 are the initial to CS transition nanoparticle numbers, and 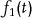 and 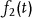 are the exponential series functions, for the first and second cycles, respectively. In Eq. (10), the nanoparticle number transitions 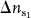 and 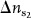 are found by spatially integrating the CS Boltzmann concentration and the exponential series are bound between zero and unity, i.e. 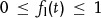, as before, l is the cycle index, 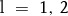. For the first cycle:


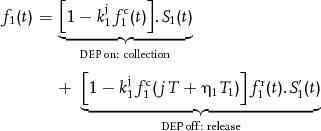
(11)

and for the second cycle:


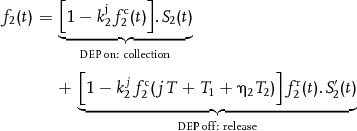
(12)

In Eq. (11), 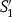 is the switch function complement for the first cycle, i.e. unity “on” for 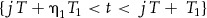 and zero elsewhere, and the same applies for 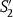 in Eq. (12). The superscripts “*c*” and “*r*” for the functions and parameters relate to the collection and release phases of each of the two cycles, which are indexed by subscripts, 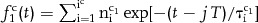, 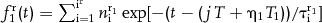, 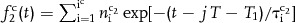, and 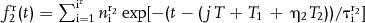. In addition, 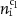 and 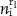 are the nanoparticle number coefficients, 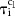 and 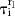 are the collection and release time constants, 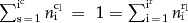 and the maximum number of components in each of the sums, *i*^c^ and *i*^r^, for convenience, is the same in both cycles. The influence of the previous cycle, in terms of nanoparticle concentrations within the capture volume, on the *j*th dual-cycle is accomplished using the collection initial condition (IC) coefficients, 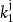 and 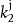. Regarding each of the two cycles, Eqs. (11) and (12), the product of the number coefficient and the IC for the *j*th cycle, 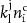, arises from the particular solution to the MDE in the form of an eigenmode expansion 19; the details are available as Supporting Information. The linear time invariant lumped parameter approximation, within each dual cycle, is reasonable for small DEP capture volumes, i.e. spatial regions where nanoparticles collect, and for pDEP, close to the electrode edges. Other examples of 1D linear MDE solutions have entailed ordinary and modified Bessel functions of the first and second kinds 21,22.

The model given by Eqs. (10)–(12) also includes the situations where high concentrations of nanoparticles interact and their presence also distorts the electric field, and hence, the DEP force. The MDE (Eq. (8)) prescribing nanoparticle motion will be nonlinear; nonetheless, simulations and experiments show that the collection and release phases can be considered separate solutions. Equations (10)–(12) remain valid except that the weight and time coefficients have different values compared to those for a linear MDE. Importantly, the DEP force may also be influenced by other electrohydrodynamic effects, such as, electro-osmosis so that the net deterministic driving motion is the effective DEP force. This means that instead of attempting to evaluate, for example, the DEP force by assigning values to all parameters in Eq. (6), the dynamics of the nanoparticle ensemble can be characterized by fitting 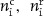 and 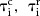 to experimental measurements and subsequently estimating the effective force.

#### 2.1.4 Dependence on dual-cycle period example

Nanoparticle excursions to and from the electrode edges depend on the strength of the DEP force that dominates and drives nanoparticles toward the electrode edges (thus achieving *n*_max_), the thermal fluctuations responsible for nanoparticles moving away from the electrode edges when the pDEP force is switched off (achieving *n*_min_); and the duration time for both of these processes. The dual-cyclic DEP collection and release of nanoparticles within a designated volume leads to an important parameter: the difference between the maximum and minimum of the number of particle for each of the two cycles. The nanoparticle number fluctuation, or amplitude, evaluated over the duration of the *j*th dual-cycle is defined:


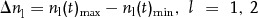
(13)

where subscripted terms “max” and “min” denote maximum and minimum particle number.

**Figure 4:**
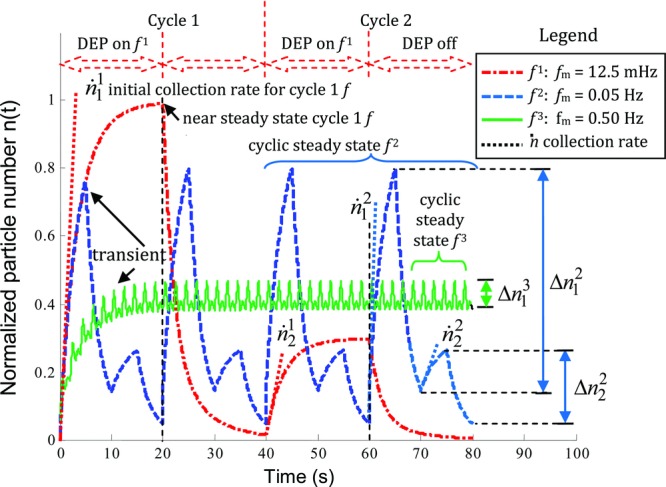
Dual-cycle DEP collection and release simulation for three modulation frequencies as listed. Normalized particle number *n*(*t*) plot for η = 0.5 (50% duty cycle), values as stated on plot (T = 1/*f*_m_) or in the text.

An example of dual-cycle DEP collection and release using the model (10)–(12) is shown in Fig. 4. For convenience, the nanoparticle initial to CS transition numbers in Eq. (10) are normalized, 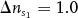 and 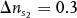 and the series has a maximum of two terms, 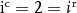. Relating to Eqs. (11) and (12), the nanoparticle number weightings used in this example are 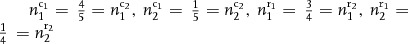 and the time constants are 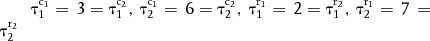
(s). This means that the shape of the second cycle is the same as the first and the only difference is the scale (0.3). The values of the weightings and time constants are based on the dynamics of submicron-sized nanoparticles, including previously reported ultralow AM DEP collection and release measurements 3, 19, 21, 22. Both duty cycles ratios are the same, 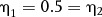. If the period of DEP switched on is sufficiently long, e.g. 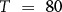 s for the first case 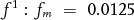 Hz where the superscript denotes the case, the nanoparticles in Fig. 4 in the first cycle are predicted initially to collect at a constant rate, 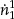 (where subscript denotes cycle), that later approaches a quasi-CS maximum for this phase of the cycle. Likewise, switching off the DEP force for the release phase, the system approaches quasi-release (phase) steady state minimum, as shown. The same applies for the second cycle except the collection rate 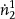 maximum is 0.3-fold smaller.

On the other hand, if the period of DEP switched on is sufficiently short, e.g. for the second case 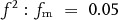 Hz or the third case 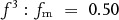 Hz, as shown, quasi-SS within either of the dual cycles is not reached since there is insufficient time for the particles to fully collect before they are released. As illustrated in Fig. 4, cases 2 and 3, an important distinction is made between transient behavior and periodic or dual-cyclic steady state (cSS), behavior. In the former case, the nanoparticle number *n* at the beginning of *j*th cycle is less than at the end, or beginning of the next cycle, 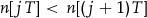, as illustrated.

In cSS, the nanoparticle number of the beginning of the *j*th first cycle and at the end of the second remains the same, 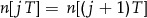. An alternative to “cSS” is “cyclostationary”—a term that describes a system with statistics that remain unchanged at periodic time points 23,24. This periodic equilibrium state implies that the IC for each cycle comprising the dual-cycle remains constant, 
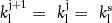
 where the superscript “s” denotes cSS and expressions for the IC, amplitude and time average can be derived. The cSS amplitudes for both cycles for case 2 in Fig. 4 show the amplitude of the first cycle is greater than the second, 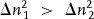 and similarly the collection rates, 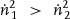, as expected. The amplitudes for case 3 that is ×10 higher modulation frequency are smaller than case 2, and as the modulation frequency increases (*T* decreases) further, the dual-cycle amplitudes in Eq. (13) become vanishingly small, 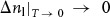. This trend is confirmed by laboratory observations of nanoparticle movement and measurements using fluorescence microscopy. For modulation frequencies above the order of 1 Hz, and for similar duty cycle ratio values, the time averages (over *T*) tends to become comparable, or larger than, the amplitudes. The pDEP collection and release processes, for each of the two cycles, become indistinguishable. Very little DEP motion or modulation, e.g. blurred fluorescence, is observed and a partial unmodulated, or constant (0 Hz), nanoparticle response appears microscopically at the electrode edges.

The transition from a prominent DEP amplitude response at ultralow *f*_m_ compared with the response being negligible at higher modulation frequencies suggests DEP “AM bandwidths.” They can be defined as the range of modulation frequencies, *f_m_*, such that at cSS:


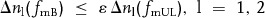
(14)

where l is the cycle index, ε is an arbitrary cut-off typically, 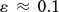, and subscripts “mB” and “mUL” denote modulation bandwidth and ultralow modulation that approaches a constant (0 Hz), in the limit 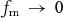.

In a typical DEP experiment, the cyclic DEP response of fluorescent nanoparticles yields a corresponding optical signal that is quantified using a microscope and video camera. A measurable parameter is the difference between the maximum and minimum, or fluorescence amplitude for each of the two cycles at cSS:


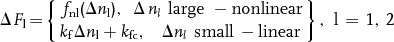
(15)

In Eq. (15), for weak DEP yielding small collections of nanospheres, the fluorescence is proportional to number where *k*_f_ is the fluorescence gradient coefficient and *k*_fc_ is the intercept constant. Eventually, further collections of nanospheres do not yield proportional increases in fluorescence and saturation occurs. The relation can be expressed by *f*_nl_ that is a nonlinear, soft limit function. The dependence on parameters at cSS is understood to be implicit, 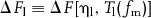. A linear or quasilinear relationship between fluorescence and nanoparticle number enables values for the two bandwidths to be estimated experimentally. The maximum modulation frequency is bound by the AM bandwidths. For example in 19, a single-cycle bandwidth was measured to be 1 Hz for 0.5 μm diameter nanospheres and this value also concurred with predictions of 1D time-dependent Fourier–Bessel series models 21,22. Clearly, there is an optimum set of modulation frequencies suitable for differential DEP collection rates suggesting that, in the case where the duty cycle ratios and cycle periods are similar to each other, 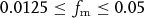 Hz is a suitable range.

### 2.2 Determining dielectric properties via collection rates

Central to determining the nanoparticle dielectric properties from the DEP collection rates is the frequency-dependent real part of the CM function. This section describes properties of the CM function and methods to fit the function to collection rate ratios. A comparison of two methods, collection rate ratios and crossover, is made in terms of nanoparticle conductivity estimates.

#### 2.2.1 Properties of the CM function

**Figure 5:**
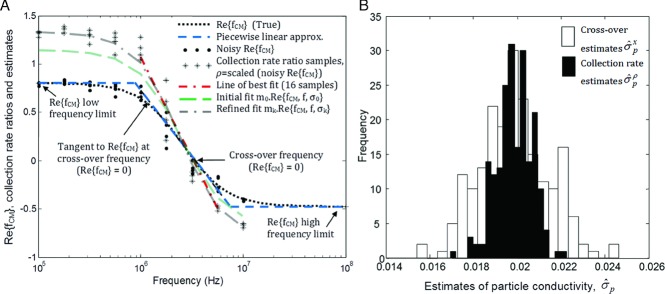
(A) Plots of the real part of CM as a function of frequency,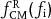, three-line piecewise approximation, and noisy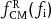 arising from noise added to the nanoparticle conductivity. The product of the noisy 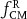 and CM scaling factor, *m*, simulates the collection rate ratio samples (“*”) that are distributed over the frequencies as nine groups of four samples. Estimation starts with initial values that are obtained by line of best fit at frequencies of a few megahertz where the collection ratios rapidly change. The two initial estimates, 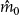
and 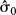, generate 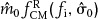 profile that is further refined using the NR iterative method, that yields estimates, 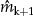 and 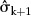 that, in turn, leads to an improved final fit, 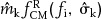 as shown. (B) Histograms of the estimated nanoparticle conductivity (S/m) from Monte-Carlo simulation, 200 trials. Collection rates show improved estimation with narrower spread or variance compared to the crossover method.

The DEP collection rates are used to determine the dielectric properties of the nanoparticles and typically for spheroidal particles, by the real part of the CM factor that describes the frequency dependent polarizability:


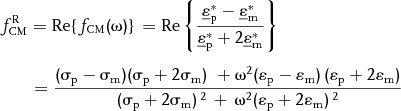
(16)

where 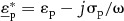 is the complex permittivity of the particle, 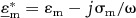 is the complex permittivity of the medium, and other symbols have been previously defined. An example of 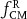 as a function of frequency is sketched in Fig. 5A using typical values for medium conductivity, 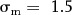
mS/m, and medium and nanoparticle permittivity, 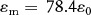 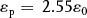 where 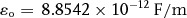 is the permittivity of free space. These values are typical for an aqueous suspension of latex nanospheres at room temperature. The nanoparticle conductivity is evaluated using a standard expression involving bulk conductivity, σ_b_, nanoparticle radius, *r*, and the surface conductance, *K*_s_, 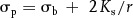. Hence, using 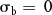, 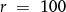 nm, 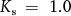
nS results in the nanoparticle conductivity being, 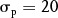 mS/m. The frequency of the crossover, *f*_x_, from pDEP to nDEP occurs when the numerator of Eq. (16) is zero:


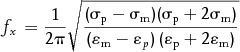
(17)

In Fig. 5A the crossover frequency is seen to be, 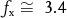
MHz. In principle, a value of the nanoparticle conductivity can be determined from a value for 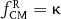 by inverting Eq. (16) and solving the quadratic relation (see Supporting Information). Typically, the positive root yields a unique solution. At the crossover, applying 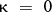, (Eq. S2.13 in Supporting Information) gives the solution 3:


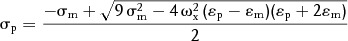
(18)

The upper and lower frequency limits of the real part of CM are labeled in Fig. 5A and are approximately constant:


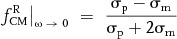
(19)


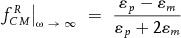
(20)

In Fig. 5A, for example, the lower and upper frequency limits are, respectively, 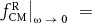 0.80 and 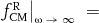 –0.48. The gradient at the crossover is given by the partial derivative of Eq. (16) with respect to the log-frequency:


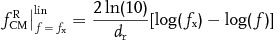
(21)

where the sum of the dielectric ratios is given by:


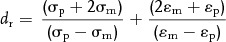
(22)

and “lin” denotes linear relation. The negative gradient at the crossover is also shown in Fig. 5A.

Thus, 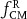 can be approximated as three lines using Eqs. (19)–(22): two horizontal lines subtending from each of the lower and upper limits, as shown in Fig. 5A, and the “diagonal” tangential to 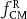 at the crossover frequency, and is thus, piecewise linear. The simple piecewise linear approximation, based on Eqs. (19)–(22), is useful for determining the dielectric properties even if it is not possible to measure the crossover frequency experimentally.

#### 2.2.2 Fitting the CM function to collection rate data

The proportional relationship between the real part of CM, that is bounded, 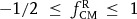 and the unbounded collection rate ratio, ρ, expressed by Eq. (09), suggests that they should be related by a scaling factor, *m*, and constant *c* such that:


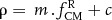
(23)

A series of collection rate ratio values, from Monte-Carlo simulations as discussed in the following sections, is shown in Fig. 5A showing the scaling factor lies between one and two. A feature of Eq. (9), verified experimentally, is that if 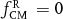 at crossover then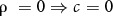. The fitting process, therefore, entails estimating from collection ratio data two parameters: (i) nanoparticle conductivity, σ_p_, and (ii) scaling factor, *m*.

The fitting process is of two steps: (i) initiation–where an initial value for σ_p_, and *m* are estimated from collection rate data, followed by (ii) refinement–where initial estimates of σ_p_, and *m*, are refined by fitting the nonlinear function, 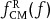 using Newton's method or a numerical method, e.g. Nelder–Mead.

Step 1: This step finds initial, rough estimates for equation parameters of the line of best-fit shown in Fig. 5A that relates the initial collection rate ratio, ρ, with log-frequency, l:


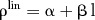
(24)

where the abscissa, α, and gradient, β, are given by standard textbook formulae, e.g. 25 for least sum of squared error (SSE) fit (see Supporting Information also). The collection rate ratio is set, for convenience, so that the baseline or “control” data points near 1 MHz, are about unity, 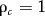 where “c” denotes “control.” This means that an initial estimate for the scaling factor is given by:


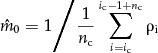
(25)

where the subscript “0” denotes initial estimate and *n*_c_ is the number of data points at the control frequency. The initial estimate for σ_p_ can be found from Eq. (24) by using the estimate of the crossover frequency line of best-fit, 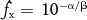, or if it is not measured, extrapolation of the line. An alternative 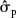 can be found by equating the log-frequency gradient of 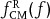 defined in Eqs. (21) and (22) with the gradient of the of the collection rate ratios line of best-fit Eq. (24):


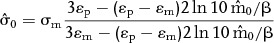
(26)

where it is understood that the subscript “p” has been replaced by “0” to signify the initial estimate. In the example shown in Fig. 5A, the parameter values for the line of best-fit, 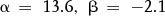 and 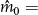
1.4 lead to 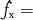
3.25 MHz, 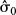 = 19 mS/m and using the gradient method,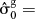 5.8 mS/m. The result of using the initial estimates 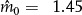 and 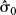 = 19 mS/m in determining the scaled real part of the CM function, 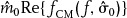 is shown in Fig. 5A. Clearly, it is close to the collection ratios but needs further refinement.

Step 2: The initial estimate of nanoparticle conductivity 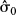 is refined by updating using the Newton–Raphson (NR) procedure, i.e. 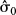 is updated to 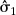 by the algorithm:


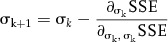
(27)

where it is understood for the first iteration (in this example) *k* = 0 and 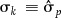 for the *k*th iteration, 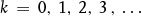. In Eq. (27), the first and second partial derivatives of the SSE are evaluated using the expression for 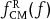 (see Supporting Information for further details). The revised conductivity, 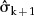, then enables estimate for the scaling factor, 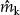 (derived in Supporting Information), to be updated to 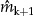:


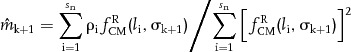
(28)

The iteration is repeated until the difference is sufficiently small, e.g. 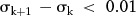
mS/m. For the collection ratios shown in Fig. 5A, the refined fit, 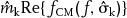, is clearly a much better fit to the collection ratio values than the initial fit.

### 2.2.3 Comparison of methods for nanoparticle conductivity estimation

In this section, estimation of nanoparticle conductivity using the crossover is compared with collection rate method. The comparison consists following two stages: (i) data simulation, and (ii) parameter estimation.

Stage 1—Simulation: The surface conductance for both methods is partly randomized by adding noise so that the *i*th sample of the nanoparticle conductivity is simulated:


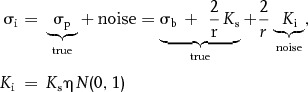
(29)

In the simulation, the dielectric parameter values in Eq. (29) are the same as before, and for clarity the true value of the nanoparticle conductivity is labeled. The additive noise factor contribution is set at 15%, η = 0.15 and the noise,*N*(0, 1), is a zero mean, unit variance random number. Since nanoparticle conductivity for the *i*th data point, σ_i_ is “noisy,” the real part of the CM function, 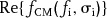 becomes noisy, as shown in Fig. 5A. The collection rate ratio, ρ_i_, is simulated as:


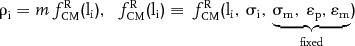
(30)

where, as before, 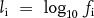 and 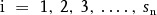 and the total number of samples, *s*_n_ = 36. The sample values are uniformly spaced across the log-frequency domain, as shown in Fig. 5A, as *n*_f_ = 9 frequency points each comprising *n*_p_ = 4 samples. The collection rate scaling factor, *m*, is set to have a random effect and a sensible value, for illustration in Fig. 5A, is found by taking the reciprocal of the root of mean of sum of squares:


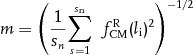
(31)

where the dependence of the fixed and randomized dielectric parameters is implicit, as in Eq. (30), so that for generating the example collection rate data shown in Fig. 5A, 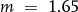.

Stage 2—Estimation: Estimation of the nanoparticle conductivity for the collection ratios is achieved by the two-step estimation process described in the previous section with relations (24)–(28). The refined estimate from the NR yielded, for the example in Fig. 5A, 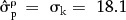 mS/m where collection rate ratio is denoted by superscript “ρ” and the scaling factor was 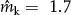. Nanoparticle conductivity values are estimated from measured crossover frequencies, *f*_x_, typically by fitting Eq. (17) for a range of medium conductivities and reading 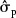, which is the equivalent of solving with the quadratic relation (18). In this comparison, for brevity, only one medium conductivity is used. Since Eq. (17) is the inverse of Eq. (18) in this context, it follows that the least SSE estimate of nanoparticle conductivity is directly given by:


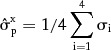
(32)

where the superscript “x” means estimated from “crossover” data.

Monte-Carlo repetition of the two-stage simulation and estimation process, 200 times, enables statistical evaluation of the estimation performance. Histograms of the estimates are shown in Fig. 5B and it is clear the spread of the estimates for using the crossover method is much wider than for the collection rate method. Summary statistics show that the mean of the estimates is comparable to each other and to the true value, 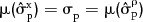. On the other hand, the variance of the estimates from the crossover method is nearly fourfold more than the variance of the estimates from the collection rate method, 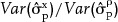 = 3.8. Since the collection method has ninefold more samples than the crossover method, a ninefold ratio would be expected from central limit theorem 25. A reduced ratio is not surprising given that the real part of the CM is nonlinear and that the high frequency limit, shown in Eq. (18), is dominated by nanoparticle and medium permittivity rather conductivity where noise has been introduced. The Monte-Carlo simulation data strongly supports the argument that collection rate measurements give a more robust and accurate measurement of the dielectric properties of the nanoparticles compared to methods which rely solely on an individual measurement of the zero force point at a number of medium conductivities (i.e. crossover measurements).

## 2.3 Experimental

The dual-cycle DEP initial collection rate ratio method for estimating nanoparticle conductivity was demonstrated experimentally using fluorescent latex nanospheres.

A signal generator (TG4001; ThurlbyThandar Instruments, Fort Worth, TX, USA) provided 2 V peak-to-peak, variable duty cycle, square wave enveloped, sinusoidal signals over a frequency range of 1–10 MHz to the microelectrodes. Pulse duration, amplitude, and applied frequencies were controlled by custom software written in LabVIEW™ 2011 (National Instruments, Austin, TX, USA).

Castellated geometry interdigitated microelectrodes with critical feature sizes of 5 μm were fabricated using standard photolithography and lift-off techniques (Fig. 1 shows an image of a microelectrode array). A 100 nm thick layer of platinum was lithographically patterned on 500 μm thick Pyrex wafers. Individual devices were cut from the wafer. The devices were mounted on Veroboard and wire bonded to the copper strips on the Veroboard allowing robust electrical connection to the signal generator. A 7 mm diameter glass ring was glued on top of the devices to form a sample chamber of defined height over the electrodes. This was then sealed with a cover-slip to prevent sample evaporation.

Ultrapure water having a resistance of 18.2 MΩcm (Purelab ultra, Elga process water, Buckinghamshire, UK) was used to prepare KCl (Sigma-Aldrich®, St. Louis, MO, USA) buffer solutions with conductivity of 2.0 × 10^−4^ S/m (Mettler Toledo, InLab® 730, Columbus, OH, USA) at room temperature. Carboxylate-modified 200 nm diameter latex spheres (Invitrogen™ Molecular Probes®, Eugene, OR, USA) with yellow-green fluorescence (505/515 nm wavelength) were washed three times in KCl buffer (2.0 × 10^−4^ S/m) and suspended in the same KCl buffer at a concentration of 4.8 × 10^10^ spheres/mL (diluted from 2% w/v stock solution). The concentration and monodispersity of the nanospheres was verified using nanoparticle-tracking analysis (NanoSight LM10, Wiltshire, UK).

The motion of the nanospheres was observed using an inverted microscope (Nikon Eclipse TS 100) with LED epifluorescent illumination (pE-2, CoolLED, UK) imaged with ×40 objective (Nikon S Plan Fluor 40 × /0.60) and recorded with a digital camera (Thorlabs USB 2.0, Newton, NJ, USA) at 10 frames/s. Videos were analyzed using bespoke software written in Matlab™ 7.14 (Mathworks, Natick, MA, USA). Nanosphere collections at the high-field gradient regions of the castellated electrodes (i.e. areas where particles collect under the influence of pDEP) were quantified by measuring the fluorescence intensity at the electrode tips. By restricting the image analysis to only the edge regions of the castellated electrodes and performing control and probe collection rate measurements, fluctuations in the background intensity were normalized (for further details of the image analysis approach, see 13, 19). Time-dependent collection profiles for each of the control and probe cycles were quantified by linear fitting to thirteen points; this proved sufficiently robust from noise and gave consistent measures of DEP strength. Linear fitting is typically performed with initial collection rates as an approximation to an exponential series for sufficiently short time intervals 4, 9–17. Collection data was also fitted to single and double exponentials and there was little quantifiable advantage using this more complicated method.

## 3 Results

**Figure 6:**
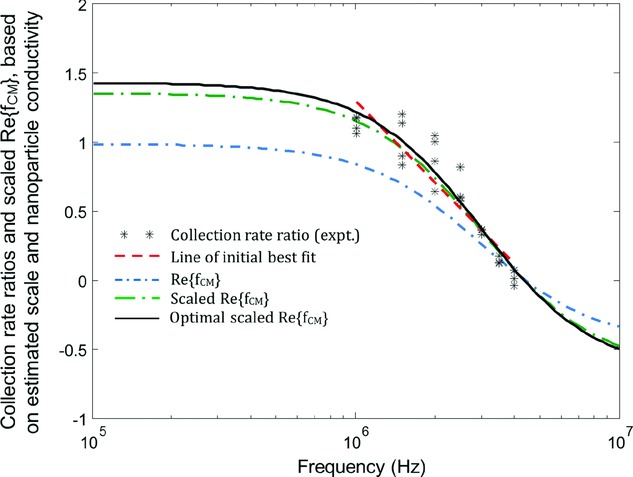
Representative initial collection rate ratio data (*) from dual-cycle DEP experiments. The line of best fit for frequencies in the range of 1–4 MHz, as shown was used to generate initial estimates for the nanoparticle conductivity and CM scaling factor, 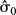 and 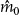. The real part of the CM, 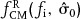 is plotted as shown along with the scaled version, 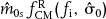. Further refinement of the estimates used the NR algorithm yielding the final 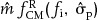 curve, as plotted, with estimates for the CM scaling factor, 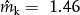 and for the nanoparticle conductivity, 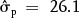 mS/m.

In each set of collection experiments, approximately 100 μL aliquots of nanosphere suspension was pipetted onto the device and sealed with a cover slip. The dual frequency collection rates comprised of a control collection pulse set at, 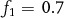 MHz and a probe pulse that ranged in carrier frequency from 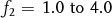 MHz. The dual-cycle consisted of equal periods, 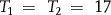 s and duty cycle ratio values, 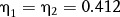 (7 s on, 10 s off), so 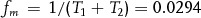 Hz. The ground-to-peak voltage, during the DEP on phase, was 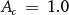
V (i.e. 2 V peak-to-peak) for all experiments. Each experiment recorded approximately five dual cycles in 170 s. Collections for the first dual cycle were transient, whereas the four remaining dual cycles exhibited cSS characteristics described in Sections 2.1.3 and 2.1.4. Thus, the choice of 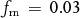 Hz achieved the following two key conditions: (i) cSS and (ii) an adequate fluorescence amplitude, 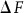, (i.e. nanoparticle fluctuation) suitable for quantification of initial collection rates. The initial collection rate ratio of the dual-cycle control and probe, for each experiment, was evaluated according to Eq. (9) and a representative data set, with four replicates for each of the seven applied frequencies, is shown in Fig. 6. Initial estimates of the nanoparticle conductivity and CM scaling factor, 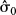 and 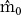, were estimated according to Eqs. (24), (26), and (31) using the line of best-fit for the applied frequencies, 1–4 MHz (28 data values) as shown. Using the initial estimate for the nanoparticle conductivity, the real part of the CM, 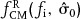 is plotted as shown along with the scaled version, 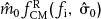. The refinement used the NR algorithm, Eqs. (27) and (28) yielding final estimates for the scaling factor, 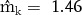 and for the nanoparticle conductivity, 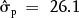 mS/m. This value is to within 8% of the expected value 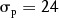 mS/m [12]. Assuming, as before, 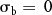, 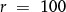 nm, then the surface conductance is estimated, 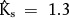
nS. The estimated crossover frequency, from Eq. (17), is 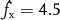 MHz, close to the expected value for low conductivity medium.

## 4 Discussion

The use of dual-cycle dielectrophoretic collection rate ratios has been demonstrated as a method for rapidly estimating the dielectric properties of nanoparticles in fluid suspension. The dual-cycle method uses a constant control frequency and a probe signal with a frequency that can be selectively switched, or hopped, throughout the range of interest. The application of a control pDEP collection cycle, preceding the probe cycle acts to mitigate against experimental variations that arise from fluctuations in localized nanoparticle concentrations. Monte-Carlo simulations demonstrate that the “more independent samples, better estimates” concept applies, thus motivating the use of collection rates compared with conventional DEP crossover measurements. DEP collection experiments demonstrated that 15 s on and 10 s off duration for each of the control and probe cycles with 1 V electrical amplitude yielded accurate collection rate data for 200 nm diameter nanospheres at 20 mHz modulation frequency and 60% duty cycle. These values are semioptimized, full optimization of parameter settings would need further investigation. The dual-cycle can be easily extended to multiple cycles, and again needing optimization and cost/benefit analysis. We have obtained similar data using 500 nm diameter nanospheres demonstrating the general applicability of the method.

An important advantage of using collection rates, compared with the crossover method, is the absence of a requirement for nDEP. Our focus in this paper has been the introduction of pDEP the dual-cycle collection ratio method and demonstration of experimental proof-of-principle. We have not explored the use of an nDEP control and probe, or indeed alternative estimation of dielectric parameters using nDEP, however, these avenues would also be exciting to explore for further application in measurement science.

The real part of the CM function was initially fitted based on a piecewise linear approximation (on the log-frequency scale) and application of least squares error to bivariate parameters, i.e. scaling and nanoparticle permittivity. There is no reason why a more complicated real part of the CM cannot be approximated by a piecewise linear model, e.g. more complex structures, such as, cells, that exhibit multiple crossovers, or, extending bivariate fitting to multivariate parameter fitting. The crossover frequency is a key feature of interest in this work, but for other investigations, the point of inflection of the real part of the CM may be of greater importance (e.g. 16) where linear fits can be made to provide initial parameter estimates.

The dual-cycle model introduced a simple and insightful mathematical framework with characteristics based on typical values for the weights and time constants that explain experimental observations, e.g. the presence of a transient response followed by cSS. The model assumes linear time invariance and correctly predicts a decrease in nanoparticle number fluctuation or amplitude with increasing modulation frequency expected of active ultra-low pass filters, and a modulation bandwidth of up to a few Hertz. Nonetheless, the modeling process could be extended further by finding empirical estimates for the weights and time constants from experimental data. Indeed, perhaps the most exciting advance in terms of estimating parameters would be at lower frequencies where other electrohydrodynamic effects begin to become significant, e.g. low-frequency electroosmosis, and corresponding parameters of these effects included in the estimation process 26. In principle, there is no limitation on the number of collection and release cycles other than the practical limitation due to data storage, sample evaporation, etc., none of which are near being limiting factors.

In conclusion, a new dual-cycle DEP fluorescence micro-spectroscopic system and mathematical framework has been developed for quantifying the dielectric properties of nanoparticles. The system entails two cycles each involving a DEP nanoparticle collection and release phase: the first cycle is a control DEP nanoparticle collection with fixed frequency, followed by release, and the collection rate in the second cycle acts as a probe since the frequency can be selectively switched. By taking the ratio of the initial collection rate of the probe with respect to the control, experimental variation between DEP collection rate experiments can be reduced. The ratios are shown to exhibit good experimental repeatability and enables probing over a range of applied frequencies. Bivariate fitting of the real part of the CM factor to the collection rate ratio values using least squares yields estimates for the scaling factor and nanoparticle conductivity. The semiautomated dual-cycle DEP system effectively allows larger sampling of nanoparticles over a range of frequencies, compared with the conventional crossover method. The benefits of the dual-cycle method are demonstrated with a Monte-Carlo simulation, which shows a narrower spread of the estimates of the nanoparticle dielectric properties. The technique was demonstrated experimentally using 200 nm latex nanospheres and shown to be in good agreement with the expected dielectric properties of these particles. The simplicity and speed of the current method makes it ideal as a method for the rapid characterization of a range of biological particles (e.g. viruses, bacteria, DNA and RNA).

## Abbreviations

AM: amplitude modulation
CM: Clausius–Mossotti
CS: collection (phase) steady state
cSS: cyclic (periodic) steady state
IC: initial condition
MDE: modified diffusion equation
nDEP: negative DEP
NR: Newton–Raphson
pDEP: positive DEP
RS: release (phase) steady state
SS: steady state
SSE: sum of squared error

## Supplementary material

As a service to our authors and readers, this journal provides supporting information supplied by the authors. Such materials are peer reviewed and may be re-organized for online delivery, but are not copy-edited or typeset. Technical support issues arising from supporting information (other than missing files) should be addressed to the authors.

## References

[1] Pethig R (2010). Biomicrofluidics.

[2] Lapizco-Encinas BH, Rito-Palomares M (2007). Electrophoresis.

[3] Morgan H, Green NG (2003). AC Electrokinetics.

[4] Hughes MP, Hughes M, Morgan H, Rixon F, Burt J, Pethig R (1998). Biochim. Biophys. Acta.

[5] Morgan H, Hughes MP, Green NG (1999). Biophys. J.

[6] Chan KL, Morgan H, Morgan E, Cameron IT, Thomas MR (2000). Biochim. Biophys. Acta.

[7] Hughes MP, Morgan H, Rixon FJ (2002). Biochim. Biophys. Acta.

[8] Ermolina I, Milner J, Morgan H (2006). Electrophoresis.

[9] Pohl HA (1978). Dielectrophoresis.

[10] Gascoyne PRC, Noshari J, Becker FF, Pethig R (1994). IEEE Trans. Indus. Appl.

[11] Talary MS, Pethig R (1994). IEE Proc. Sci. Meas. Tech.

[12] Bakewell DJ, Morgan H (2001). IEEE Trans. Dielectr. Electr. Insul.

[13] Bakewell DJ, Morgan H (2004). Meas. Sci. Tech.

[14] Bakewell DJ, Morgan H (2006). IEEE Trans. Nanobiosci.

[15] Henning A, Bier FF, Holzel R (2010). Biomicrofluidics.

[16] Giraud G, Pethig R, Schulze H, Henihan G, Terry JG, Menachery A, Ciani I, Corrigan D, Campbell CJ, Mount AR, Ghazal P, Walton AJ, Crain J, Bachmann TT (2011). Biomicrofluidics.

[17] Hawkins BG, Kirby BJ (2011). Anal. Chem.

[18] Carlson AB, Crilly PB, Rutledge JC (2002). Communication Systems: An Introduction to Signals and Noise in Electrical Communication.

[19] Bakewell DJ, Chichenkov A (2012). J. Phys. D: Appl. Phys.

[20] Kreyszig E (1968). Advanced Engineering Mathematics.

[21] Bakewell DJ (2011). J. Phys. D: Appl. Phys.

[22] Bakewell DJ, Chichenkov A (2012). IEEE Trans. Nanobiosci.

[23] Papoulis A (1984). Probability, Random Variables, and Stochastic Processes.

[24] Gardiner CW (1985). Handbook of Stochastic Methods for Physics, Chemistry, and the Natural sciences.

[25] Walpole RE, Myers RH (1978). Probability and Statistics for Engineers and Scientists.

[26] Castellanos A, Ramos A, Gonzalez A, Green NG, Morgan H (2003). J. Phys. D: Appl. Phys.

