# Supplementary material for: Dual-cycle dielectrophoretic collection rates for probing the dielectric properties of nanoparticles
Source: Electrophoresis. 2013 Mar 6;34(7):987–99. doi: 10.1002/elps.201200422 (PMC3770930; doi:10.1002/elps.201200422)
Supplement: Supplementary file 1 [file elps0034-0987-SD1.doc]

**Dual-cycle dielectrophoretic collection rates for probing the dielectric properties of nanoparticles**

**David J. Bakewell^1^ and David Holmes^2^**

^1^Department of Electrical Engineering and Electronics, University of Liverpool, Brownlow Hill, Liverpool, L69 3GJ, UK. Email: d.bakewell@liv.ac.uk

^2^London Centre for Nanotechnology, University College London, 17-19 Gordon Street, London, WC1H 0AH, UK. Email: david.holmes@ucl.ac.uk

# Supplementary Information

***S1:DEP nanoparticle transport model***

Consider the DEP ‘on’ phase of the cycle. The FPE for nanoparticle concentration is given by

$\frac{\partial c(\underline{x},t)}{\partial t} = - \vec{\nabla}\cdot\vec{J}(c(\underline{x},t)) = \underbrace{-\frac{1}{\zeta}\vec{\nabla}(c(\underline{x},t)F_{DEP}(\underline{x}))}_{DEP} + \underbrace{\frac{k_{B}T}{\zeta}\vec{\nabla}\cdot\vec{\nabla}c(\underline{x},t)}_{Diffusion}$ (S1.1)

and the nanoparticle flux is where $F_{DEP}(\underline{x})$ is the DEP force, *k_B_T* is the Boltzmann temperature, and *ζ* is the nanoparticle dynamic drag coefficient. Expanding the concentration in a series product of separate functions [20],

$c(\underline{x},t)=\sum_{m = 0}^{\infty} g_{m}(\underline{x})h_{m}(t)$ (S1.2).

Substituting (S1.2) into (S1.1) yields two separate, linear Ordinary Differential Equations (ODEs). The first ODE has unity order with a General Solution (GS) for each eigenmode *m*,

$h_{m}(t)=e^{-t/\tau_{m}}$ (S1.3)

where$\tau_{m}$is an integration constant. The second ODE is second order

(S1.4)

Application of boundary conditions (BCs) and IC enables the GS of (S1.4) to give a Particular Solution (PS). The BCs, e.g. [21,22] can be reflecting so that no nanoparticles are allowed to enter or escape the system. Integrating over spatial volume yields a series expression for the nanoparticle number

$n(t) = \int_{V} c(\underline{x},t) d\underline{x}=\sum_{m = 0}^{\infty} \int_{V} g_{m}(\underline{x})d\underline{x}h_{m}(t)=n_{0}+\sum_{m = 1}^{m_{x}} \underbrace{\int_{V} g_{m}(\underline{x})d\underline{x}}_{number} e^{- t/\tau_{m}}$ (S1.5)

where the *m* = 0^th^ term, *n*_0_, is steady state where $t\to\infty$ and the infinite series is truncated to *m_x_* depending on the tolerated error.

Including the exponential series in each phase of a cyclic process needs consideration of the IC at the start of each cycle. The integrand in (S1.5) can be split, the IC is approximated to be uniform so its integral can be evaluated separately. This approximation is satisfactory when *V* is sufficiently small with respect to the entire chamber volume, and enables independence of the eigenmode summing index. Typically, application of the boundary conditions and IC means the evaluated integral in (S1.5) is negative, so that the integral becomes

(S1.6)

where $\hat{c}(\underline{x},0)$ is the normalized concentration, or probability density, such that $0 < k < 1$ and *N_m_* represents nanoparticle number. Substituting (S1.6) into (S1.5) and noting that *n*_0_ is independent of the IC

$n(t) = n_{0}[1-k\sum_{m = 1}^{m_{x}} n_{m} e^{- t/\tau_{m}}]$ (S1.7)

where$n_{m}= N_{m}/n_{0}$is the nanoparticle number coefficient, normalized for convenience. Equation (S1.7) forms the Fourier series solution that can be applied for both nanoparticle collection and modified for release. To achieve self-consistency in simulations the truncation error is set to zero by requiring

$\sum_{m = 1}^{m_{x}} n_{m} = 1$ (S1.8)

The steady state, *n*_0_, is replaced by the initial to collection steady state transition, Δ*n_s_* and the eigenmode index is replaced by a general index. A simpler argument applies for the DEP ‘off’ nanoparticle release phase of the cycle. Using the switching function, the exponential series in (S1.7) can be set up to model the collection and release phases of the *j*^th^ dual-cycle. Adding superscripts to the variables to denote the two DEP ‘on’ and ‘off’ phases, subscripts to denote the cycle number (within the dual-cycle), etc., (10) – (12) follow.

***S2: Estimation of nanoparticle parameters***

*Expressions for estimate initiation:*

An initial estimate for nanoparticle conductivity uses the line of best fit. This can be derived by taking the sum of the squares of the residual error, $e_{i}$, (SSE) between the ratios of collection rate (simulated) data, $\rho_{i}$, and the line to be fitted,

$SSE=\sum_{i=1}^{s_{n}} e_{i}^{2}=\sum_{i=1}^{s_{n}} [\rho_{i}-(\beta l_{i} + \alpha)]^{2}$ (S2.1)

The abscissa, *α*, is found by finding the minimum SSE, i.e. setting the partial derivative of SSE, with respect to that parameter, to zero, $\partial SSE/\partial\alpha=0$ and yields

$\hat{\alpha}=\frac{1}{s_{n}}\sum_{i=1}^{s_{n}} \rho_{i}-\frac{\beta}{s_{n}}\sum_{i=1}^{s_{n}} l_{i}= \bar{\rho}-\beta\bar{l}$ (S2.2)

Similarly, the gradient, *β*, is found by finding the minimum SSE, setting the partial derivative to zero,

$\frac{\partial SSE}{\partial\beta}=-2\sum_{i=1}^{s_{n}} [\rho_{i}-(\beta l_{i} + \alpha)]l_{i}=0$ (S2.3)

and leads to the standard textbook formula for least squares fit

$\hat{\beta}=\frac{s_{n}\sum_{i=1}^{s_{n}} \rho_{i}l_{i}-\sum_{i=1}^{s_{n}} \rho_{i}\sum_{i=1}^{s_{n}} l_{i}}{s_{n}\sum_{i=1}^{s_{n}} l_{i}^{2}-\left(\sum_{i=1}^{s_{n}} l_{i}\right)^{2}}$ (S2.4)

*Expressions for estimating refinement*:

The sum of the squares of the residual error,$e_{i}$, (SSE) between the ratios of collection rate (simulated) data, $\rho_{i}$, and predicted data from the product of the scaling factor, *m*, and real part of the CM function for the *i*th sample at the log-frequency, $l_{i}=\log_{10}f_{i}$,

$SSE=\sum_{i=1}^{s_{n}} e_{i}^{2}=\sum_{i=1}^{s_{n}} [\rho_{i}-mf_{CM}^{R}(l_{i})]^{2}$ (S2.5)

The optimal value for the scaling factor is found by finding the partial derivative of SSE with respect to that parameter and setting to zero to find the minimum,

$\frac{\partial SSE}{\partial m}=-2\sum_{i=1}^{s_{n}} [\rho_{i}-mf_{CM}^{R}(l_{i})]f_{CM}^{R}(l_{i})=0$ (S2.6)

which leads to

$\hat{m}=\sum_{i=1}^{s_{n}} \rho_{i}f_{CM}^{R}(l_{i})/\sum_{i=1}^{s_{n}} [f_{CM}^{R}(l_{i})]^{2}$ (S2.7)

Minimizing SSE with respect to the nanoparticle conductivity, the first and second partial derivatives are

$\partial_{\sigma_{k}}SSE=\frac{\partial SSE}{\partial\sigma_{k}}=2\sum_{i=1}^{s_{n}} \rho_{i}. \partial_{\sigma_{k}}f_{i}^{R}-2m\sum_{i=1}^{s_{n}} f_{i}^{R}. \partial_{\sigma_{k}}f_{i}^{R}$ (S2.8)

and

$\partial_{\sigma_{k}, \sigma_{k}}SSE=\frac{\partial^{2}SSE}{\partial\sigma_{k}^{2}}=2\sum_{i=1}^{s_{n}} \rho_{i}. \partial_{\sigma_{k},\sigma_{k}}f_{i}^{R}-2m\sum_{i=1}^{s_{n}} [f_{i}^{R}. \partial_{\sigma_{k},\sigma_{k}}f_{i}^{R} + (\partial_{\sigma_{k}}f_{i}^{R})^{2}]$ (S2.9)

where standard notation is understood $f_{i}^{R}\equiv f_{CM}^{R}(l_{i}), \partial_{\sigma}f_{i}^{R}\equiv\frac{\partial f_{CM}^{R}(l_{i})}{\partial\sigma_{p}} \text{and} \partial_{\sigma,\sigma}f_{i}^{R}\equiv\frac{\partial^{2}f_{CM}^{R}(l_{i})}{\partial\sigma_{p}^{2}}$ for the *k*th iteration of the conductivity estimate. The partial derivatives of the Clausius-Mossotti (CM) factor, after some algebra, are given by

$\partial_{\sigma}f_{i}^{R}\equiv\frac{\partial f_{CM}^{R}}{\partial\sigma_{p}}= \frac{3 \sigma_{m} \sigma_{p}^{2}+a\sigma_{p}+b}{[(\sigma_{p}+2\sigma_{m}) ^{2} + \omega^{2}(\varepsilon_{p}+2\varepsilon_{m}) ^{2}]^{2}}$ (S2.10)

and

$\partial_{\sigma,\sigma}f_{i}^{R}\equiv\frac{\partial^{2}f_{CM}^{R}}{\partial\sigma_{p}^{2}}= \frac{-6 \sigma_{m} \sigma_{p}^{3}+-3a\sigma_{p}^{2}+c\sigma_{p}+d}{[(\sigma_{p}+2\sigma_{m}) ^{2} + \omega^{2}(\varepsilon_{p}+2\varepsilon_{m}) ^{2}]^{3}}$ (S2.11)

where $a = 6 [2\sigma_{m}^{2} + (\sigma_{p}+2\varepsilon_{m})\varepsilon_{m}\omega^{2}]$, $b = 3\varepsilon_{m} [4\sigma_{m}^{2} + (4\varepsilon_{m}^{2}-\varepsilon_{p}^{2}) \omega^{2}]$, $c = 18\sigma_{m} [-4\sigma_{m}^{2} + (\varepsilon_{p}^{2}-4\varepsilon_{m}^{2}) \omega^{2}]$ and $d = 6\omega^{2}(\varepsilon_{p}+2\varepsilon_{m}) [6\varepsilon_{p}\sigma_{m}^{2} + \varepsilon_{m}(\varepsilon_{p}+2\varepsilon_{m})^{2} \omega^{2}]-48\sigma_{m}^{4}$.

These expressions are then inserted into the Newton-Raphson method for solving a nonlinear equation. Note that the nanoparticle conductivity for a single value of the real part of the CM factor is given by

$f_{cm}^{R}=\mathrm{Re}\{f_{CM}(\omega)\} =\frac{(\sigma_{p}-\sigma_{m})(\sigma_{p}+2\sigma_{m}) +\omega^{2}(\varepsilon_{p}-\varepsilon_{m}) (\varepsilon_{p}+2\varepsilon_{m})}{(\sigma_{p}+2\sigma_{m}) ^{2} + \omega^{2}(\varepsilon_{p}+2\varepsilon_{m}) ^{2}}\equiv\kappa$ (S2.12)

where it assumed the value is real and bounded, $-1/2 < \kappa< 1$. Re-arranging into a quadratic dependence yields a solution with one positive real root

$\sigma_{p}=\frac{-\sigma_{m}(1-4\kappa)+\sqrt{\sigma_{m}^{2}(1-4\kappa)^{2}-4(1-\kappa)g}}{2(1-\kappa)}$ (S2. 13)

where$g=\omega^{2}[\varepsilon_{p}^{2}(1-\kappa)+\varepsilon_{p}\varepsilon_{m}(1-4\kappa)-2\varepsilon_{m}^{2}(1+2\kappa)]-2\sigma_{m}^{2}(1+2\kappa)$ . Setting $\kappa= 0$for the cross-over condition and re-arranging, yields (18).
